# Aiming to increase birth weight: a randomised trial of pre-pregnancy information, advice and counselling in inner-urban Melbourne

**DOI:** 10.1186/1471-2458-6-299

**Published:** 2006-12-10

**Authors:** Judith Lumley, Lisa Donohue

**Affiliations:** 1 Mother and Child Health Research, La Trobe University, 251 Faraday St Carlton, Victoria 3053, Melbourne, Australia; 2 Key Centre for Women's Health in Society, University of Melbourne, Level 1, 305 Cardigan St, Carlton, Victoria 3053, Melbourne, Australia; 3 Western Hospital, Gordon St Footscray, Victoria 3011, Melbourne, Australia

## Abstract

**Background:**

In the 1980s there was substantial interest in early pregnancy and pre-pregnancy interventions to increase birth weight and reduce preterm birth. We developed an inter-pregnancy intervention, implemented in a randomised controlled trial, to be provided by midwives at home soon after women's first birth.

**Methods:**

MCH nurses invited women to take part during their home visit to new mothers. Women's contact details, with their permission, were passed to the study midwife. She had a randomisation schedule to which women's names were added before she met the women or their partners. All women recruited had a home visit from the study midwife with a discussion of their first pregnancy, labour and birth and the postpartum experience. Women in the intervention arm received in addition a pre-pregnancy intervention with discussion of social, health or lifestyle problems, preparation and timing for pregnancy, family history, rubella immunisation, referrals for health problems, and a reminder card. The primary outcome was defined as a birth weight difference in the second birth of 100 g (one-sided) in favour of the intervention. Additional data collected were gestational age, perinatal deaths and birth defects. Analyses used EPI-INFO and STATA.

**Results:**

Intervention and comparison groups were comparable on socioeconomic factors, prior reproductive history and first birth outcomes. Infant birth weight in the second birth was lower (-97.4 g,)) among infants in the intervention arm. There were no significant differences between intervention and comparison arms in the proportion of women having a preterm birth, an infant with low birthweight, or an infant with a birth weight <10^th ^percentile. There were more adverse outcomes in the intervention arm: ten births <32 weeks), compared with one in standard care, and more infants with a birth weight <2000 g, 16 compared with two in standard care

**Conclusion:**

As the primary outcome was envisaged to be either improved birth weight or no effect, the study was not designed to identify the alternative outcome with confidence. Despite widespread support for pre-pregnancy interventions to improve maternal and perinatal health, this first randomised controlled trial of a multi-component intervention provided at home, did not have a beneficial outcome.

## Background

At the time this project was initiated (1980–85) there was substantial interest in the idea of pre-pregnancy – or pre-conception – counselling and care. Pre-pregnancy care was discussed in three contexts. The first was routine advice, counselling and care around rubella immunisation, nutrition, smoking, alcohol consumption, and exercise for *all *women, prior to conception and thus prior to antenatal care [1,2] The second was provision of advice and counselling on specific issues for women perceived to have *increased risks *of an adverse pregnancy outcome; women with recurrent miscarriage, a prior perinatal death, a prior infant with low birthweight or an infant with a major birth defect [3]. The third was care of women with *known risks *to the fetus or themselves from maternal health problems such as diabetes, epilepsy, heart disease, renal disease, or from indicated treatment [4].

The rationale, then and now, was that many factors affecting fetal health and survival, or maternal health, are present well before the first antenatal visit, often before conception, limiting options for effective intervention in pregnancy [5]. Other considerations were that assistance and support for modifying behaviours such as smoking might be more effective prior to pregnancy, given the increased anxiety that may occur in early pregnancy. Before assessment of gestational age by ultrasound scanning became common, there were also concerns that a 'natural' cycle and a reliable date for the last menstrual period needed to be re-established before conception; thus women were advised to replace hormonal contraception or intrauterine devices with barrier methods for several months before trying to conceive.

### Pre-pregnancy Information and Counselling Service (PPIS)

The setting in which the trial was planned was a newly established pre-pregnancy service (PPIS) in inner urban Melbourne [6]. It was initiated by two obstetricians from a University Department of Obstetrics and Gynaecology, in association with a Community Health Centre, and funded within a new, national, community health programme. The primary role of the PPIS was to provide a 'universal' walk-in service locally, and a secondary school health education component. It also accepted referrals and provided a telephone information service. One fulltime, experienced, midwife, subsequently increased to 1.5 positions, staffed the PPIS, with support from the first author. The PPIS self-referral and telephone service attracted in the main well educated and socially advantaged women, who were not living in the locality. This finding, together with the policy that evaluation was to have a key role in services funded by the new community health program, contributed to the development of the pre-pregnancy intervention trial.

Objectives of the PPIS trial

1. To recruit women into a randomised trial of pre-pregnancy advice/counselling in a community setting where mothers were at higher risk of a poor outcome. The three local areas had substantial numbers of recent migrants, refugees, single parents, families with a low income and families in public housing

2. To assess the effectiveness of the intervention in terms of the birthweight of the second child, as the most sensitive single indicator of fetal wellbeing.

3. To incorporate the intervention, including data collection and follow-up, into the routine work of community health nurses/midwives and Maternal and Child Health Nurses (MCHN) to be able to assess the feasibility of implementing the intervention more widely if it were effective.

Local MCHNs were key partners in the trial. They provided a universal service funded by local government and State government, from postnatal hospital discharge, at that time more than five days after birth for the majority of women, to school entry. In Victoria, all births are notified by the hospital of birth to the MCH Centre closest to the woman's residence, with a statutory home visit by the MCHN in the first week after hospital discharge. Over 95% of women attend the MCH Centre in the first six months after birth [8]. Consultations were held with the Director of Maternal and Child Health (Health Department of Victoria), the Regional Infant Welfare Advisors, Infant Welfare staff in municipalities close to the Community Health Centre, the Administrator and Board of Management of the Community Health Centre, and the Central Health Interpreter Service, in the planning phase. At these meetings we discussed the trial rationale, the intervention, planned recruitment and follow-up, seeking input on how these could be incorporated into the standard working of each of the services. There was strong support for the proposed trial across all those consulted.

## Methods

### Design

The study design was a randomised controlled trial, with individual randomisation. It was designed to be able to identify a mean birth weight difference of ≥ 100 g in the intervention arm. There was no Data Monitoring Committee and no stopping rules were defined.

### Participants and recruitment procedures

All women attending local MCH Centres with their first child were eligible for inclusion in the trial. Each MCHN discussed the project briefly with all eligible women and asked permission to give the PPIS midwife their contact details. The PPIS midwife collected women's names and addresses weekly from the MCH nurses, and recorded whether an interpreter would be needed, and for what language. Women's names were then entered into the trial logbook where the sequence of allocations (derived from random number tables by the first author and utilising balanced block randomisation in blocks of two, four or six) was already listed. Thus allocation occurred before the PPIS midwife had met the woman. The PPIS midwife contacted women whose details had been provided and arranged a home visit to confirm participation. There was a Central Health Interpreter Service in Melbourne at that time making it possible to book an interpreter for a specific language for the home visit, so that a significant proportion of immigrant women could be included.

Recruitment began in May 1982 and ended in July 1991. The timing of recruitment was in the early months after the first birth, to ensure that the women's own health was recognised as a key factor, as it has been in more recent interventions [7]. Follow-up continued to the end of 1994.

### The intervention

#### Comparison group

All women recruited received a home visit from the PPIS midwife with a discussion of their first pregnancy, labour and birth and the postpartum experience. Any questions asked by the women were answered.

#### Intervention group

Women randomised to receive the intervention received a pre-pregnancy health intervention that consisted of:

1. Identification of any current social, health or lifestyle problems.

2. Discussion of timing, planning and preparation for the next pregnancy

3. Offers of referral for any specific problem identified (e.g. to a dietician, relaxation group, physiotherapist, family planning clinic, general practitioner) all available at the Community Health Centre or nearby, or at a local hospital clinic; linkage with appropriate community resources (e.g. language-specific play-group) and networks.

4. Taking a family/genetic history and arranging a referral if necessary.

5. Arranging for rubella immunisation if not immune

6. Discussion of the points summarised on a WAIT, STOP, and GO reminder card. The card was headed *Signs to follow before pregnancy*, and designed to mimic traffic lights:

 WAIT (Orange)

• For 3 months after using the pill or loop

• For 3 months after German measles vaccination

• Until you really want another child

 STOP (Red)

• Cigarettes

• Alcohol

• Other over the counter drugs and medications

 Go (Green)

• To the doctor when you have missed two periods

• Regularly for antenatal checks

• Ring us if you have any questions or problems

The card included the name and address of the PPIS and the telephone number.

The commonest issues raised by women in the intervention arm at this time were choice of contraceptive method (n = 274), the 'ideal' interval between children (262), smoking (202), rubella (199), diet (177), operative birth (175), alcohol (172), breastfeeding (170), sex since the birth (166), caring for the baby (121), the decision to have another child (102) and postnatal depression (76).

### Sample size, primary outcome and secondary outcomes

The study was designed to identify a difference of ≥ 100 g in the primary outcome – the mean birth weight of the second children, in favour of the intervention arm of the trial (α = 0.05, β = 0.80. one-sided), calculated as 394 in each arm. Loss to follow-up was predicted to be at least 20%, making the total recruitment goal 950 women [9] Secondary outcomes were gestational age at birth, preterm birth grouped as 20–27 weeks, 28–31 weeks, and 32–36 weeks; low birth weight (<2,500 g), perinatal deaths and birth defects. Birth weights less than the sex-specific 10^th ^percentile were added later. All twins were included in the birth weight analysis [10]. They were excluded from birth weight < 10^th ^percentile as the standards for this categorisation were derived from singleton births only [11].

### Data collection and analysis

Information about the previous pregnancy and birth was hand written on A5 cards at the first home visit by the PPIS midwives, and updated at later contacts. Where possible, follow-up of the subsequent birth took place at a planned home visit, but telephone and – letters were also used, especially when women had moved outside the local area. Most participants provided details for at least one close relative as a secondary contact. When these processes were inadequate, follow-up data on the outcome of the second pregnancy and birth were sought from the referring MCHN and, if the woman had moved to another part of the State, from the MCHN in the new area. Transfer of the data to computer did not occur until 1995. EPI INFO [12] was used for data checking and cleaning.

Data analysis was carried out using EPI-INFO and STATA version 8 [13]. Characteristics of the parents and the first child were described and tabulated. Intervention and comparison groups were compared in terms of the first and second child's birth weight (mean and standard deviation), the proportions of infants with low birth weight, preterm birth and in preterm subgroups, and among singletons the proportions with a birth weight below the 10th percentile for gestational age and sex. Given the very small number of infants who died or had birth anomalies these are described in full but not formally compared. One analysis that was not pre-specified but prompted by study findings compared the time interval from the first birth to the conception of the second infant in the two arms of the trial.

## Results

Table 1 shows the flow of participants through the trial: 1688 were approached, 109 refused (65 in the intervention arm, 44 in standard care), and 1579 agreed to take part. Many of those who refused were defined as *de facto *refusals since they had agreed to take part, and provided contact details, but did not keep appointments, were not at home for up to three planned visits, behaved in a threatening way – or their partner did – or refused entry to the PPIS midwife. When participating women had their first visit a further 11% were found to be ineligible (see Table 1). A further 84 had already moved out of the area at the time of the first attempted contact, 63 were already pregnant and 29 already had two or more children, something not known to the MCH service. More women from the intervention arm (157) than in the standard care arm (128) were lost or ineligible, leaving 1403 active participants. Women subsequently lost to the study were those who decided not to have another child (n = 31), usually for reasons of severe parental ill health; those who had not had a second pregnancy by 1995 (222), and 364 who were lost to follow-up. Fewer women were lost to follow-up from the intervention arm (173) than from the standard care arm (191) at this stage making the final participants 392 in the intervention arm and 394 in the comparison arm.

The socio-demographic and past reproductive history characteristics of the study families are summarised in Table 2. The population recruited was typical of inner-urban Melbourne at that time, in terms of the proportion of women born outside Australia (more than a third), the proportion born in a country where English was not the primary language (a quarter), and the other countries of birth most commonly represented (Vietnam and Greece). The occupations of fathers were similar in the two arms [14], as were the specific Local Government Areas of the families' residence at recruitment. Prior reproductive problems and mode of delivery at the first birth were also similar in intervention and comparison groups. The two groups did not differ in the duration of breastfeeding, the type of contraception used, or the interval between the intervention and the birth of the second child.

Table 3 summarises the perinatal outcomes of the first births. The mean birth weights differed by only 39 g in intervention and comparison arms and the standard deviation was the same in both arms. The proportion with an infant of low birth weight (<2,500 g) was similar in the two arms, (5% and 4%). The proportion of infants with a birth weight below the 10^th ^percentile using sex-specific tables was also similar (16% and 13%). The mean gestational age was identical in the two arms. There was a small difference in rates of preterm birth, 6.5% in the intervention and 4.6% in the comparison arm There were no differences in very preterm births (<32 weeks gestation). There were no perinatal deaths and no infants with major birth anomalies among the participants' first births. The infants included four sets of twins, one in the intervention arm and three in the comparison arm.

Table 4 summarises the perinatal outcomes post-intervention. At the second birth the primary outcome measure was a significant difference in mean birth weight, but the outcome was not in the predicted direction. Babies in the intervention arm were lighter by 97 g. There were no significant differences in mean gestational age, though the standard error was larger in the intervention arm. The proportion with a birth weight below the 10^th ^percentile using sex-specific tables did not differ significantly in the two arms, being 11% in the intervention arm and 8% in the comparison arms. There were more infants with birth weights below 1500 g and below 2000 g in the intervention arm, and a marked difference in births <32 weeks gestation, with ten births in the intervention arm and one in the comparison arm The second births included four sets of twins, two in the intervention and two in the comparison arms.

There were seven known perinatal death among the second births, four associated with birth defects and three infants born extremely preterm, including one set of twins. The perinatal mortality was 8.9/1000 births. The perinatal mortality in the State of Victoria for 1983–92 was 8.8/1000 births [15]. Infants known to have birth anomalies included two with anencephaly, one with major multiple malformations (not specified), one with multiple heart defects and an absent right kidney, one with a ventricular septal defect, one with aniridia, one with Hirschsprung's disease and one with a fetal heart defect, not otherwise described, and congenital heart block. Five infants with a birth anomaly were in the intervention arm, two in the comparison arm, a combined prevalence of 8.8/1000. In 1983–94 the overall prevalence of birth anomalies in singletons in Victoria, including stillbirths and terminations of pregnancy for an identified malformation was 3.1% [16].

An additional analysis prompted by the greater number of very preterm births in the intervention arm identified a shorter intervention to conception interval among very preterm births in that arm. Their mean interval was 82 weeks (SD 37) compared with a mean of 103 weeks (SD 63) for all births in the intervention arm, and 101 weeks (SD 64) for all births in the comparison arm.

## Discussion

The PPIS trial was designed at the same time as a number of other very different pregnancy interventions designed to increase mean birth weight, and to reduce low birth weight and preterm birth through the provision of social support or enhanced nutrition [17-23]. None of the others implemented their intervention package *before *pregnancy but most aimed to recruit women relatively early in pregnancy. Four of these five trials and a recently reported additional trial [24] identified unexpected adverse outcomes, particularly extremely preterm births, or late terminations of pregnancy, i.e. findings similar to those in the PPIS trial. One factor which all of those trials had in common was that the intervention was provided by someone who was not the usual provider of antenatal care. In the one trial where there were no such unexpected adverse outcomes the supportive intervention was provided at home by midwives, a pattern within usual maternity care in the UK, where the trial was carried out [17]. Midwives provided the intervention in the PPIS trial at home but this was not usual care in Australia.

Three hypotheses to explain the possible adverse outcomes of the PPIS trial came out of discussions with colleagues in Australia and Canada. The first was that the intervention itself might have heightened parental stress and anxiety about perinatal outcomes, with stress and anxiety contributing to very preterm birth [25,26]. The second was that there might have been an increased risk of conception at the extremes of the fertile period, giving rise to unplanned conception or less than ideal implantation, if women and their partners had given up their usual method of contraception for an alternative one. The shorter intervention to conception interval associated with the very preterm births in the intervention arm might be a marker for this risk factor. The third hypothesis was that if women and their partners took a very positive approach to the full intervention package it might have improved their overall health to a point where fetuses with a birth anomaly or with poor placentation were actually sustained *in utero *for a longer period of time, with a shift from miscarriage to very preterm birth.

Alternative explanations are that detailed discussions of family health increased perceptions of risk, or led to difficulties between the parents or difficulties with their families of origin. Recommendations to stop smoking, alcohol and over the counter drugs might have been another problematic piece of advice. It is also possible that the 'medicalisation' of such a human and family-focussed event as pregnancy and birth was in itself harmful. We could not totally rule out a contribution from selective participation in the study at the initial home visit: 65 women in the intervention arm, compared with 44 in standard care, opted out of the study. Overall, there were no significant differences in participation between the two arms.

*Informed consent *of participants was sought through the three-fold process outlined in the Methods: discussion of the trial by the MCHN, verbal agreement by the woman to have her details given to the PPIS midwife and subsequent contact by the midwife to make an appointment and follow-up with a visit. Consent of the Community Health Centre, and the Maternal and Child Health service was based on a series of consultations held with each group, before the trial was implemented to describe the study and respond to queries, comments and criticisms. The Community Health Centre did not have a Human Research Ethics Committee and there was no equivalent body at that time in the then Health Commission of Victoria. When the project was awarded National Health and Medical Research Council research funding to complete the follow-up of second births (1992) we were required to provide written information about the study to all subsequent participants and to seek formal Ethics approval by the Board of the Community Health Centre. The Board co-opted an Ethics Committee Member from one of the nearby teaching hospitals to assist in this process.

*Potential limitations of the intervention *included the absence of advice to take periconceptional folate. In 1981–2 there was still marked disagreement about the benefits and risks of vitamins and folate before conception and in the first months of pregnancy. Publication of the proceedings of a workshop held in late 1982 for experts involved in recent or planned research on the prevention of neural tube defects, discussed the available evidence and its shortcomings and we were influenced by the evident uncertainty and disagreement [27]. By the time the four trials of periconceptional supplementation were published [28-31] recruitment to the PPIS trial was virtually complete.

Another potential limitation was the quality of the evidence on the effect of alcohol use before pregnancy. Several US papers had reported adverse effects on the fetus of even extremely low maternal consumption of alcohol, in early pregnancy or immediately prior to pregnancy, as well as effects of paternal alcohol use around the time of conception [32,33]. Despite the biological implausibility of some of these effects and the relatively small numbers of people in the studies we did advise stopping alcohol consumption before and during pregnancy. Contemporary population-based data on alcohol and tobacco use, collected in early pregnancy in another Australian state (Tasmania) did not show adverse effects of alcohol on fetal growth or preterm birth until maternal consumption was reported at more than two standard drinks a day [34], a level of drinking reported by 0.4% of Tasmanian women. Subsequent research in large cohorts has not confirmed the early US studies [35,36].

*Limitations of the trial *included slow recruitment. It occurred at half the rate planned, extending the duration of the trial and making it harder to maintain local interest and Maternal and Child Health Nurses' enthusiasm. Another factor slowing recruitment was staff turnover [37]. A common work pattern at the time was moving to a new position after two years, to get a broad experience in community settings. Each new staff member needed a familiarization and training period: many were unfamiliar with randomised trials at the time of recruitment.

A greater limitation was the research team's lack of recognition that the women participating were of lower, rather than higher risk of adverse pregnancy outcomes. The decision to recruit women through existing services for mothers and infants failed to take into account the fact that women who had adverse outcomes in their first pregnancy (a perinatal death, an infant with a major congenital anomaly or a very preterm infant) would be under-represented. Mothers whose infants had died would not be part of MCH services and mothers of infants requiring on-going care from hospitals or specialist services were likely to have been using those services, or visits to private paediatricians, for routine health advice and support as an alternative to their local MCHN. None of the first-born infants included in the PPIS trial was born before 30 weeks gestation.

More than 50% of participants moved house before the birth of their second child, making follow-up a much larger part of the midwives' role than originally planned [38]. The average interval between the first and second births was a mean of 39 months compared with the predicted two years. This extended the time required for follow-up and made follow-up more difficult.

*Other pre-pregnancy interventions *are sparse. A recent systematic review of preconception interventions [39] found only one randomised trial [40] which used an opportunistic strategy, recruiting women who had a negative pregnancy test, explaining the trial, inviting participation, and randomising by tossing a coin. All participants were given a preconception risk survey. Although an average of over eight risks per woman were identified, and more than half the women made another visit to the service in the next year, there was no difference between intervention and usual care arms in the proportion of risks addressed. A recently reported trial recruited women who had given birth spontaneously before 34 weeks gestation, to be randomised four months after that birth to receive either oral azithromycin 1 g twice (4 days apart) plus 750 g of sustained-release metronidazole daily for seven days, or identical – appearing placebos, repeated every 4 months until the subsequent pregnancy. The intervention did not reduce subsequent preterm birth with these authors also concluding that the intervention might be associated with a shorter gestational age and lower birth weight [41].

*The PPIS study strengths *are that it remains the only completed trial of a broad pre-pregnancy intervention. It demonstrated that it was feasible to test a pre-pregnancy intervention using a 'gold standard' design within socially disadvantaged communities, including women on low incomes and culturally diverse women, incorporating the intervention into an existing population-wide service. It provided a model for joint action between four community health services. It also identified issues relevant to the planning of future trials. Data on the high proportion of participants who turned out to be ineligible or lost to the study are likely to be relevant to other population based pre-pregnancy interventions.

## Conclusion

Despite wide-ranging support for pre-pregnancy interventions to improve maternal and perinatal outcomes [42] none of the published trials so far, nor this trial, has identified beneficial effects.

## Competing interests

The author(s) declare that they have no competing interests.

## Authors' contributions

JL designed the study, carried out the data analysis and drafted the paper. LD managed the data collection, followed the participants, and contributed to the paper. Both authors read and approved the final manuscript.

## Pre-publication history

The pre-publication history for this paper can be accessed here:



## Figures and Tables

**Table 1:** Recruitment, refusals, eligibility, and later study losses

**Recruitment, randomisation and losses**	**Intervention**	**Comparison**
Women approached	842	846
Refused	**-65**	**-44**
Agreed to participation, recruited and randomised	777	802
Ineligible post randomisation		
Moved before 1^st ^contact	-44	-40
Pregnant before 1^st ^contact	-33	-30
Baby was a 2^nd ^or later child	-15	-14

		
**Ineligible and/or refused prior to trial**	**Total -92**	**Total -84**

Eligible and consenting to participation	685	718
Subsequent loss to the study		
Decision not to have another child	-12	-19
No 2^nd ^pregnancy by end 1994	-108	-114
Lost to follow-up	-173	-191

		
**Ineligible or lost to follow-up of those eligible and consenting**	**Total -293**	**Total -324**

**Study completion**	**392**	**394**

**Table 2:** Characteristics of the participants

	Intervention: N = 392		Comparison: N = 394	
**Maternal age**	N	%	N	%
<20	10	2.6	18	4.6
20–24	81	20.9	70	17.9
25–29	127	32.4	144	36.9
30–34	135	34.2	129	33.1
35–39	36	9.2	27	6.9
≥ 40	0	-	2	0.5
Missing data	3	-	4	-
**Region of birth (Mother)**				
Australia	254	64.5	279	70.8
New Zealand/UK	23	5.9	13	3.3
Europe	28	7.1	24	6.1
Asia/Middle East	75	19.1	71	18.0
Africa	6	1.5	4	1.0
America	5	1.3	3	0.8
Missing data	1	-	0	-
**Occupation (Father)**				
Professional	16	4.1	14	3.6
Proprietor/Manager	24	6.1	15	3.8
Office and sales worker	89	22.7	111	28.2
Farmer	87	22.2	83	21.1
Skilled worker	60	15.3	53	13.5
Semiskilled worker	72	18.4	79	20.0
Unskilled worker	42	10.7	39	9.9
Missing data	2	-	0	-
**Local Government area**				
A	181	46.1	190	48.2
B	120	30.6	112	28.4
C	66	16.8	72	18.3
Other	25	5.6	20	5.1
**Prior problems**				
Infertility	31	7.9	36	9.1
Prior miscarriage ≥ 1	37	9.4	43	10.9
Prior termination ≥ 1	43	11.0	36	9.1
1^st ^birth unplanned	105	26.8	115	29.2
1^st ^birth unwanted	11	2.8	7	1.8
**First birth**				
Spontaneous vaginal*	180	46.1	206	52.3
Forceps	118	30.0	115	29.2
Vacuum	3	0.8	2	0.5
Elective caesarean	15	3.6	9	2.3
Emergency caesarean	48	12.3	40	10.2
Missing data	28	7.2	22	5.6

* includes 4 vaginal breech births in each arm

**Table 3:** Perinatal outcomes of the 1^st ^birth (pre-intervention)

	**Intervention **N = 392	**Comparison **N = 394	
**Birth weight**			
Mean (SD), g	3296 (509)	3335 (504)	Difference -39 g,
Missing data	(3)	(2)	
Birth weight <10^th^percentile*	43 (11%)	32 (8%)	OR (exact) 1.39 [0.84,2.33]
Low Birth weight			
<1000 g	0	0	
1000–1499	1	2	
1500–1999	4	4	
2000–2499	14	11	
All low birthweight	19 (4.9%)	17 (4.3%)	
	365	373	
Missing data	(8)	(6)	
All births	392	396	
**Gestation (weeks)**			
Mean (SD)	39.6 (1.7)	39.6 (1.7)	
Preterm birth (<37 weeks)	25 (6.5%)	17 (4.6%)	OR (exact) 1.14 (0.56–2.34)
Preterm birth			
20–27 weeks	0	0	
28–31 weeks	1	1	
32–36 weeks	24	16	
All preterm gestations	25 (6.5%)	17 (4.6%)	'OR 1.51(0.77. 2.98)
All with gestations ≥ 37 weeks	361	371	
Missing data	(6)	(6)	
All gestations	392	394	

*All twins excluded

**Table 4:** Perinatal outcomes of the 2nd birth (post-intervention)

	**Intervention **N = 392	**Comparison **N = 394	
**Birth interval (months)**			
Mead (SD)	40.0 (30)	38.1 (26)	F 0.89, p 0.35; unequal variance in birth interval
Missing	(2)	(1)	
**Birth weight**			
Mean (SD)	3403 (509)	3500 (504)	Difference -97 g
Missing data	(3)	(2)	
Low birth weight (<2500 g)	25/393	14/394	OR (exact) 1.85 [0.91, 3.91]
Birth weight <10^th^percentile*	40/378 (11%)	31/382(8%)	OR (exact) 1.14 [0.55, 2.38]
Low birth weight			
<1000 g	6	0	
1000–1499	3	0	
1500–1999	7	2	
2000–2499	9	12	
All with birth weight (≥ 2,500 g	367	380	
Missing data	(3)	(2)	
All births	395	396	
**Gestation**			
Mean (SD)	39.3 (2.2)	39.5 (1.5)	
Preterm birth (<37 weeks)	24 (6%)	17 (4%)	OR (exact) 1.44 (0.73, 2.91)
Preterm birth			
20–27 weeks	4	0	
28–31 weeks	6	1	
32–36 weeks	14	16	
All with gestations ≥ 37 weeks	366	374	
Missing data	(2)	(3)	
All gestations	392	394	

*All twins excluded.

**Table 5:** Reporting details for randomised trials

PAPER SECTION And topic	Item	Description	Reported on Page #
*TITLE & ABSTRACT*	1	How participants were allocated to interventions (*e.g*., "random allocation", "randomized", or "randomly assigned").	4
*INTRODUCTION *Background	2	Scientific background and explanation of rationale.	3
*METHODS *Participants	3	Eligibility criteria for participants and the settings and locations where the data were collected.	3–4
Interventions	4	Precise details of the interventions intended for each group and how and when they were actually administered.	4–5
Objectives	5	Specific objectives and hypotheses.	4
Outcomes	6	Clearly defined primary and secondary outcome measures and, when applicable, any methods used to enhance the quality of measurements (*e.g*., multiple observations, training of assessors).	5
Sample size	7	How sample size was determined and, when applicable, explanation of any interim analyses and stopping rules.	5
Randomization – Sequence generation	8	Method used to generate the random allocation sequence, including details of any restrictions (*e.g*., blocking, stratification)	4–5
Randomization – Allocation concealment	9	Method used to implement the random allocation sequence (*e.g*., numbered containers or central telephone), clarifying whether the sequence was concealed until interventions were assigned.	4
Randomization – Implementation	10	Who generated the allocation sequence, who enrolled participants, and who assigned participants to their groups.	4–5
Blinding (masking)	11	Whether or not participants, those administering the interventions, and those assessing the outcomes were blinded to group assignment. When relevant, how the success of blinding was evaluated.	n/a
Statistical methods	12	Statistical methods used to compare groups for primary outcome(s); Methods for additional analyses, such as subgroup analyses and adjusted analyses.	6
RESULTS Participant flow	13	Flow of participants through each stage (a diagram is strongly recommended). Specifically, for each group report the numbers of participants randomly assigned, receiving intended treatment, completing the study protocol, and analyzed for the primary outcome. Describe protocol deviations from study as planned, together with reasons. n/a	7, 22, Table 1
Recruitment	14	Dates defining the periods of recruitment and follow-up.	8
Baseline data	15	Baseline demographic and clinical characteristics of each group.	6, Table 2
Numbers analyzed	16	Number of participants (denominator) in each group included in each analysis and whether the analysis was by "intention-to-treat". State the results in absolute numbers when feasible (*e.g*., 10/20, not 50%).	392 and 394, I-T-T
Outcomes and estimation	17	For each primary and secondary outcome, a summary of results for each group, and the estimated effect size and its precision (*e.g*., 95% confidence interval).	Tables 3 and 4
Ancillary analyses	18	Address multiplicity by reporting any other analyses performed, including subgroup analyses and adjusted analyses, indicating those pre-specified and those exploratory.	n/a
Adverse events	19	All important adverse events or side effects in each intervention group.	8
DISCUSSION Interpretation	20	Interpretation of the results, taking into account study hypotheses, sources of potential bias or imprecision and the dangers associated with multiplicity of analyses and outcomes.	8–10
Generalizability	21	Generalizability (external validity) of the trial findings.	9–10
Overall evidence	22	General interpretation of the results in the context of current evidence.	9–10
